# Genome-Wide Identification and Analysis of Small Nucleolar RNAs and Their Roles in Regulating Latex Regeneration in the Rubber Tree (*Hevea brasiliensis*)

**DOI:** 10.3389/fpls.2021.731484

**Published:** 2021-10-26

**Authors:** Xiang Chen, Zhi Deng, Dingwei Yu, Xiaofei Zhang, Zewei An, Wenguan Wu, Qun Liang, Xiao Huang, Huasun Huang, Han Cheng

**Affiliations:** Key Laboratory of Biology and Genetic Resources of Rubber Tree, Ministry of Agriculture and Rural Affairs, Rubber Research Institute, Chinese Academy of Tropical Agricultural Sciences, Haikou, China

**Keywords:** snoRNAs, *Hevea brasiliensis*, non-coding RNA sequencing, laticifer-abundant, latex regeneration

## Abstract

Small nucleolar RNAs (snoRNAs) are a class of conserved nuclear RNAs that play important roles in the modification of ribosomal RNAs (rRNAs) in plants. In rubber trees, rRNAs are run off with latex flow during tapping and need to be regenerated for maintaining the functions of the laticifer cells. SnoRNAs are expected to play essential roles in the regeneration of rRNAs. However, snoRNAs in the rubber tree have not been sufficiently characterized thus far. In this study, we performed nuclear RNA sequencing (RNA-seq) to identify snoRNAs globally and investigate their roles in latex regeneration. We identified a total of 3,626 snoRNAs by computational prediction with nuclear RNA-seq data. Among these snoRNAs, 50 were highly expressed in latex; furthermore, the results of reverse transcription polymerase chain reaction (RT-PCR) showed the abundant expression of 31 of these snoRNAs in latex. The correlation between snoRNA expression and adjusted total solid content (TSC/C) identified 13 positively yield-correlated snoRNAs. To improve the understanding of latex regeneration in rubber trees, we developed a novel insulated tapping system (ITS), which only measures the latex regenerated in specific laticifers. Using this system, a laticifer-abundant snoRNA, *HbsnoR28*, was found to be highly correlated with latex regeneration. To the best of our knowledge, this is the first report to globally identify snoRNAs that might be involved in latex regeneration regulation and provide new clues for unraveling the mechanisms underlying the regulation of latex regeneration.

## Introduction

Small nucleolar RNAs (snoRNAs), which range from 50 to 300 nt in size (Kiss, 2002), are a distinct class of non-coding RNAs (ncRNAs) that play important roles in ribosomal RNA (rRNA) maturation by guiding ribose *2′*-*O*-methylation or pseudouridylation modification of the precursor rRNA (Tollervey and Kiss, 1997; Bachellerie et al., 2002; Watkins and Bohnsack, 2012). Generally, there are two subclasses of snoRNAs: C/D box snoRNAs and H/ACA box snoRNAs (Dieci et al., 2009). The C/D box snoRNAs are characterized by conserved 5′-end C box sequence (RUGAUGA) and 3′-end D box sequence (CUGA), and some contain less conserved internal copies of each box, known as C’ box and D’ box, respectively (Watkins et al., 2000; Kiss, 2002; Lui and Lowe, 2013). This subclass of snoRNAs guides methylation at the *2′*-hydroxyl group (*2′*-*O*-ribose methylation) in rRNAs, transfer RNAs (tRNAs), and small nuclear RNAs (snRNAs) (Brown et al., 2003). Typically, H/ACA box snoRNAs have ACA motifs at the 3′-end and common hairpin-hinge-hairpin-tail secondary structures with H box (ANANNA) in the hinge region (Kiss-László et al., 1998; Lui and Lowe, 2013). H/ACA box snoRNAs guide pseudouridine modification in rRNAs and snRNAs, which convert uridine into pseudouridine (Ψ) (Tollervey and Kiss, 1997; Watkins and Bohnsack, 2012).

However, several previous studies have shown that many snoRNAs known as “orphan snoRNAs” have typical characteristics of snoRNAs but lack complementarities to snRNA, rRNA, tRNA, or other reported RNAs (Bachellerie et al., 2002). In addition to canonical modification functions, some snoRNAs were involved in the process of pre-mRNA alternative splicing. For example, compelling evidence has been gathered for the SNORD115 family of snoRNAs, which were found to be deleted in individuals with Prader–Willi syndrome (PWS), to direct alternative splicing of the serotonin receptor subtype (5-HT2CR) pre-mRNA (Kishore and Stamm, 2006; Doe et al., 2009; Kishore et al., 2010). In addition, snoRNAs can be further processed into small ncRNA (sno-miRNAs), which are almost similar in function to the miRNAs (Lee et al., 1993; Ender et al., 2008; Kawaji et al., 2008; Taft et al., 2009; Burroughs et al., 2011). Understanding the emerging relationship between snoRNAs and RNA silencing may reveal more about the role of RNA in gene regulation (Lui and Lowe, 2013). Additionally, snoRNAs regulate chromatin assembly and are involved in cellular response to stress (Nicoloso et al., 1996; Chow and Heard, 2009; Michel et al., 2011; Schubert et al., 2012). Thus, these findings suggest that the potential roles of snoRNAs should be extensively investigated.

To date, snoRNAs have not been extensively studied in plants, and only a few studies have reported snoRNAs in *Arabidopsis thaliana*, *Oryza sativa* (rice), and other plants. To date, 295 and 544 snoRNA genes were discovered in *A. thaliana* and rice, respectively (Liang-Hu et al., 2001; Chen et al., 2003; Liu et al., 2012). Recently, the emergence of high-throughput next-generation sequencing (NGS) technology has facilitated the rapid identification of ncRNAs, such as snoRNAs, and the determination of their expression profiles at a genome-wide level in different species. Global identification and analysis of snoRNAs in rice showed 433 snoRNA candidates, including 125 previously un-annotated snoRNAs based on their structural features and common motifs (Liu et al., 2012). In maize, high-throughput sequencing was performed to identify a total of 169 ncRNAs, including 70 snoRNAs. Moreover, target site analysis revealed that 22 snoRNAs can guide to 38 sites of *2′*-*O*-methylation and pseudouridylation modification of rRNAs and snRNAs (Li et al., 2018). A combination of high-throughput sequencing and massive analysis of complementary DNA (cDNA) ends (MACE) was used to identify the complete tomato pollen sncRNAome, namely, miRNAs, snoRNAs, and tRNAs, and the regulatory network of these small RNAs was integrated into heat stress response in different stages of the development of tomato pollens (Bokszczanin et al., 2015).

The rubber tree (*Hevea brasiliensis*) is a tropical perennial crop originating in the Amazon basin and is the most economically important tropic crop in many tropical countries that provides the largest renewable natural rubber (NR) for the industry (Lau et al., 2016). The rubber tree latex is the cytoplasm of laticifers, and the latex exudes when the bark is tapped. A major part of the latex consists of rubber particles, lutoids, and other cytoplasmic components; in addition, it contains a small amount of laticifer nuclei (Shearman et al., 2014). High turgor pressure in the laticifers results in the release of the cytoplasm from the laticifers (Dickenson, 1965). However, biotic and abiotic stresses, namely, drought, salinity, temperature, mechanical wounding, and pathogen attacks, are the main factors affecting the production of latex (Jaspers and Kangasjärvi, 2010). During tapping, rRNA and proteins are run off from the laticifers and need to be re-synthesized before the next tapping to ensure the continuous yield of latex (McMullen, 1962). rRNAs are the fundamental component of ribosomes, which are the machinery for protein synthesis (Yamashita et al., 2016). Therefore, rRNA maturation, during which proteins required to maintain the biological activity in laticifers are synthesized, is considered to be the first step in latex regeneration. rRNAs are first transcribed as pre-rRNAs, and only after *2′*-*O*-methylation or pseudouridylation modifications they are processed into mature rRNAs (Tupý, 1988). Therefore, snoRNAs are expected to play important roles in latex regeneration. To date, a few snoRNAs have been identified in *H. brasiliensis*, and their functional roles in *H. brasiliensis* are largely unknown (Gébelin et al., 2012); therefore, deep sequencing should be performed to identify and analyze the snoRNAs and will contribute to a better understanding of the mechanism underlying the regulation of their expression (Chow et al., 2007).

In this study, we constructed and sequenced a non-coding RNA population ranging from 50 to 300 nt from the rubber tree leaves and latex samples for global screening and identifying potential functional snoRNAs in the rubber tree. We identified a total of 3,626 snoRNAs by this extensive analysis, and showed that 50 of them were highly expressed in latex; the expression of 31 out of the 50 snoRNAs in latex was confirmed by reverse transcription polymerase chain reaction (RT-PCR). The results of the analysis of the correlation between snoRNA expression and latex yield showed that 13 laticifer-abundant snoRNAs were highly associated with latex yield and total solid content (TSC). *HbsnoR28* was highly correlated with latex regeneration in the depletion test. Thus, our results improve the understanding of latex metabolism and regeneration in the rubber tree.

## Materials and Methods

### Preparation of the Plant Material

Five-year-old mature rubber trees (*H. brasiliensis* cv. Reyan7-33-97) were maintained at the experimental plantation of the Rubber Research Institute, Chinese Academy of Tropical Agricultural Sciences (Danzhou, Hainan, China). The leaves from the first umbrella were harvested, and the latex was collected using the conventional S/2 d/3 (half spiral cut tapped at the third daily frequency) tapping system. Samples from 10 tapped trees were combined into one biological replicate. Each experiment was conducted using at least three replicates. Total latex production was measured using the conventional approach of S/2 d/3 and was weighed as TSC after air drying at 60°C.

### Isolation of Nuclear RNA and Construction of Complementary DNA Libraries of Non-coding RNAs

The leaves were cut into 0.5- to 1-cm-wide strips. The samples were treated with cellulase (R-10; Solarbio^®^, Beijing, China) to produce protoplasts (Tang et al., 2007). The protoplasts were washed five times with a washing buffer (154 mM NaCl, 125 mM CaCl_2_, and 5 mM KCl) and preserved at −80°C. The nuclei were isolated using a nuclear isolation buffer [NIB; 10 mM N-2-hydroxyethylpiperazine-N-2-ethane sulfonic acid (HEPSE), 0.8 M sucrose, 5 mM MgCl_2_, 5 mM ethylenediamine tetraacetic acid (EDTA), pH 7.6, and then 0.07% β-mercaptoethanol after autoclaving]. The nuclei of laticifer cells present in the latex were centrifuged at 16,000 rpm for 2 h under 4°C using an ultracentrifuge (Sovall^TM^ LYNX 4000; Thermo Fisher Scientific^TM^, United States). We collected only the precipitate containing most of the lutoid and a small amount of laticifer nuclei. The pellet was washed two times by violent vortexing and resuspending in the NIB. Then, the TRIzol method (Invitrogen^TM^, United States) was used to isolate the nuclear RNAs according to the protocol of the manufacturer. The concentrations of samples were measured using an ultra-microspectrophotometer (NanoDrop2000; Thermo Fisher Scientific^TM^, United States). Furthermore, rRNAs were isolated using Ribo-Zero rRNA Removal Kits (Plant Leaf, MRZPL116; Illumina^®^, United States) according to the standard protocol.

The sequencing libraries were constructed as previously described (Deng et al., 2006). Briefly, RNA fractions of 50–500 nt in length were isolated by polyacrylamide gel electrophoresis (PAGE) and sequentially ligated to the 5′ and 3′ end adaptors. Then, the adapter-ligated RNAs were reverse transcribed using primers with partial complementarity to the adaptors. The cDNAs with both end adaptors were subsequently sequenced using HiSeq2500 (Illumina, United States).

### Data Analysis and *de novo* Transcriptome Assembly

Raw reads were trimmed using Trim_Adaptor script^1^ with the minimum clip length fixed at six nucleotides. The clean paired-end reads were assembled using the Trinity program (Trinityrnaseq_r20131110) with the following parameters: seqType, fastaq; JM, 500G; minimum k-mer value, 1; CPU, 32 (Grabherr et al., 2011). The clean reads were mapped onto the reference genome of the rubber tree (Tang et al., 2016) with Tophat2 v2.1.0. to calculate the mapping location using default parameters. For differential expression calculation, RSEM v1.2.8 was used according to protocols previously described (Cheng et al., 2018).

### Identification of Small Nucleolar RNAs and Prediction of Modification Sites in the Ribosomal RNAs Sequence

Small nucleolar ribonucleic acid candidates were identified using snoScan 0.9b (Lowe and Eddy, 1999) and snoSeeker v1.1 (Yang et al., 2006) on the basis of the assembled transcriptome. On the basis of the known modification sites in *A. thaliana*, *O. sativa*, and other model organisms, we predicted the putative modification sites on the rRNA sequence according to consensus rRNA sequence between the rubber tree and the model plant organisms. Guide snoRNA candidates were identified by comparing them with rRNA sequences in *H. brasiliensis* with the following parameters: C/D box snoRNA > 14 and H/ACA box snoRNA > 40. If the snoRNA candidates were located in the exon, the threshold value would be set higher (C/D box snoRNA ≥ 20, H/ACA box snoRNA ≥ 45). To remove rRNAs and other structural gene sequences from the snoRNA candidates, the candidates were aligned to the eukaryotic rRNA sequences (download from ftp://ftp.ncbi.nlm.nih.gov/blast/db/), and the published rubber tree EST sequences (Ko et al., 2003; Cheng et al., 2016) using BLASTn v2.8.1 RSEM v1.2.8 (Li and Dewey, 2011) and EBSeq v1.32.0 (Leng et al., 2016) software were used to analyze the expression levels of snoRNA candidates in the leaves and latex with the false discovery rate (FDR) value set at 0.05.

### Identification of Putative Small Nucleolar RNA Target Sites

Methylation sites in the rRNAs were computationally predicted using the CDSeeker program by scanning for complementary regions of the snoRNAs from corresponding 5.8S, 18S, and 28S sequences in *H. brasiliensis*. The first nucleotide of the D or D’ box was included in the guide sequence, because this position may participate in the formed duplex. One mismatch, two GU base pairs, and no bulges were allowed in the duplex between the guide and the rRNA region containing the target sequence. Typically, the H/ACA box snoRNAs are distinguished by the presence of an ACA motif at the 3′-end and a common hairpin-hinge-hairpin-tail secondary structure with the H box (ANANNA) in the hinge region.

### Analysis of the Expression of Small Nucleolar RNAs

Total RNAs were extracted from the leaves and latex using the Trizol method. We reverse-transcribed 2 μg of total RNA to cDNA using a SuperScript III reverse transcriptase kit (Invitrogen, United States) according to the protocol of the manufacturer. All the expression analyses were conducted with three biological replicates. *HbI5I* was used as the internal reference for gene expression analyses. Semi-quantitative PCR was performed using the following conditions: 95°C for 5 min, followed by 30 cycles of denaturing at 95°C for 15 s, annealing at 60°C for 30 s, and elongation at 72°C for 30 s. About 10 μl of the PCR product was analyzed by agarose gel electrophoresis. Quantitative RT-PCR (qRT-PCR) was performed to detect the expression level of each snoRNA in the hybrid population. Specific primers for respective snoRNAs (Supplementary Table 4) were designed using Primer 5, and all of the amplified fragments were sequenced for target verification. The qRT-PCR reaction was performed on the LightCycler 2.0 System (Roche Diagnostics, Penzberg, Germany) using the SYBR Green premix kit (TaKaRa, Dalian, China) according to the instructions of the manufacturer. The relative abundance of transcripts was calculated using the Light Cycler Relative Quantification Software. Spearman’s correlation was performed with adjusted TSC (TSC/C) and qRT-PCR results using R scripts.

### Latex Regeneration Using an Insulated Tapping System

We designed an insulated tapping system (ITS) in this study (Figures 6A,B). Briefly, a 50-cm-tall bark was insulated by girding the trunk at a height of 50 and 100 cm. The girding was carefully performed to cut the yellow layer, where, the laticifers are abundant while causing no damage to the inner watery layer, which transports nutrients. In the ITS, the tapping will exude the latex only from the dedicated 50-cm bark, and the transportation of nutrients will not be affected.

**FIGURE 1:**
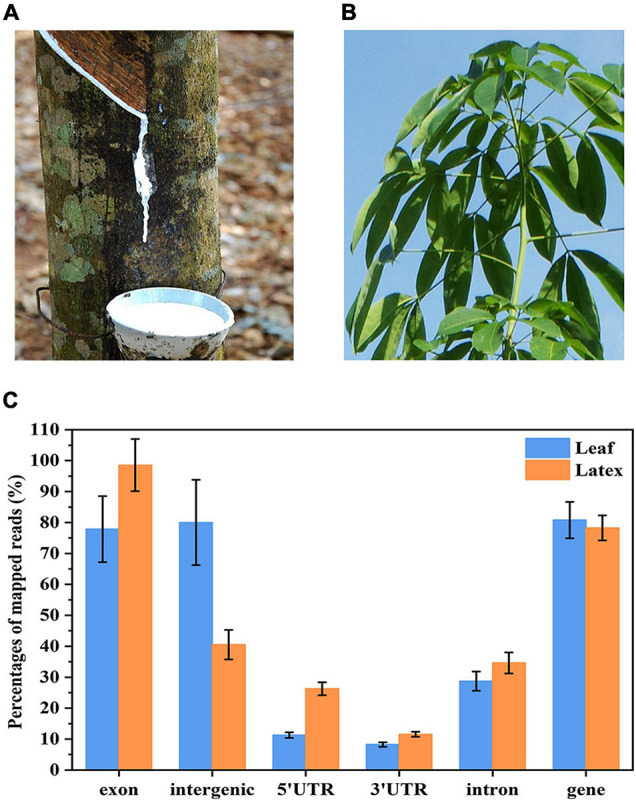
Ribonucleic acid (RNA) sequencing of the nuclei prepared from **(A)** latex and **(B)** leaves of the rubber tree. The reads were mapped onto the rubber tree genome and **(C)** percentages of the mapped reads on each region were calculated. As the read is 150 nt, one reads may map onto several regions.

**FIGURE 2:**
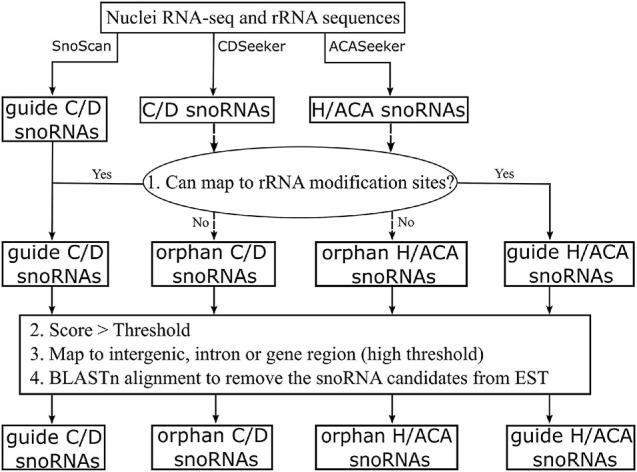
Flowchart of identification and prediction of small nucleolar RNAs in the rubber tree.

**FIGURE 3:**
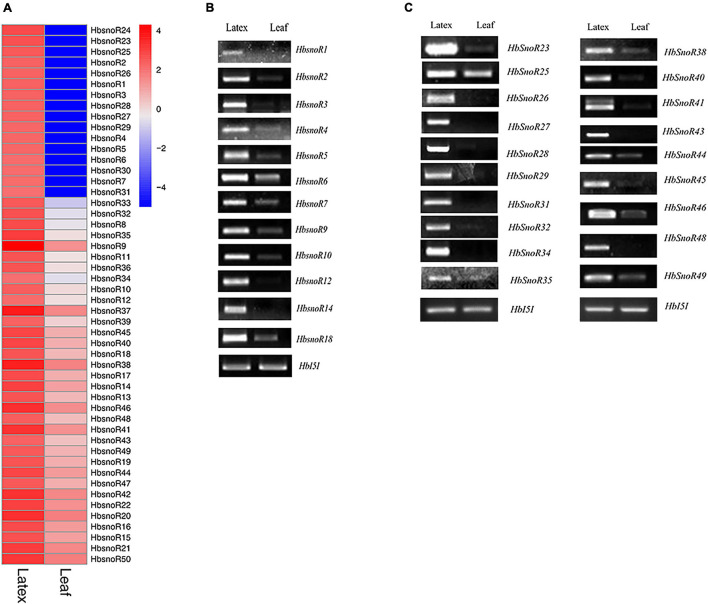
Identification of latex-abundant small nucleolar RNA (snoRNAs). **(A)** Heatmap of differential expressed (DE) snoRNA expression between latex and leaf by comparative transcriptomic analysis. **(B)** Reverse transcription polymerase chain reaction (RT-PCR) validation of 12 latex-abundant C/D subfamily snoRNAs. **(C)** RT-PCR validation of 19 latex-abundant H/ACA subfamily snoRNAs.

**FIGURE 4:**
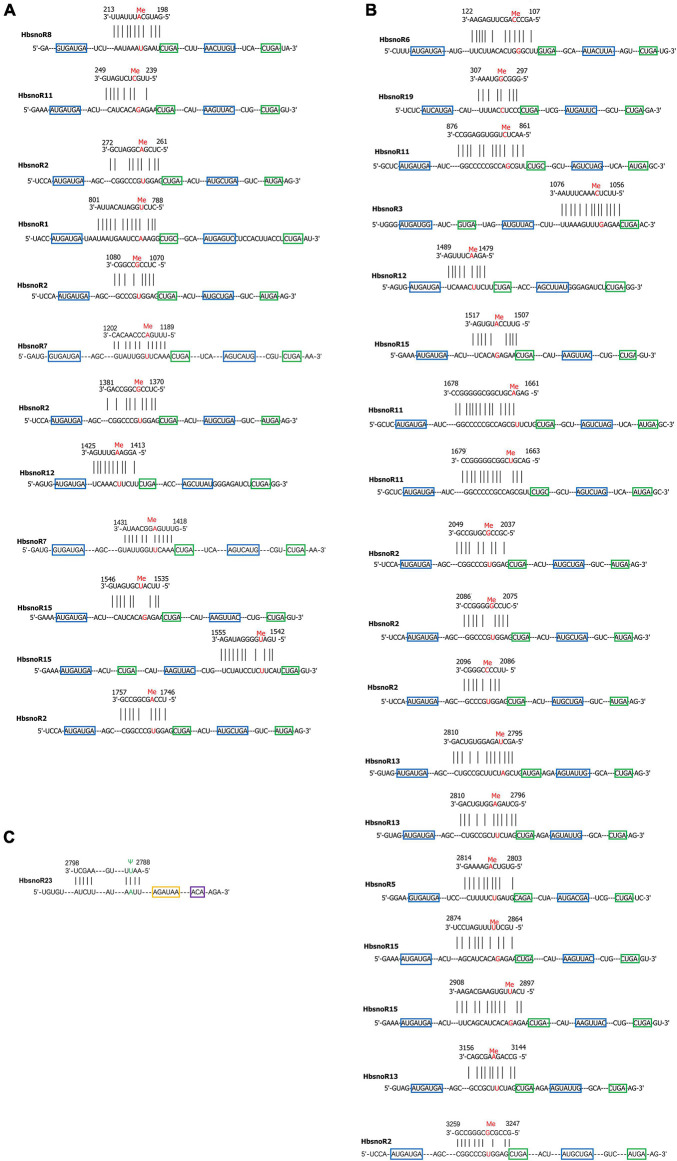
**(A)** Predicted targeting sites of C/D subfamily small nucleolar RNA (snoRNAs) on **(A)** 18S and **(B)** 28S rRNA. The nucleotides under *2′*-*O*-methylation were indicated in red color. The C box was shown in blue, while the D box was in green. **(C)** Predicted targeting sites of H/ACA subfamily snoRNAs on 28S rRNA, the nucleotide under pseudouridylation was indicated in green color. H box is shown in yellow and the ACA box in purple.

**FIGURE 5:**
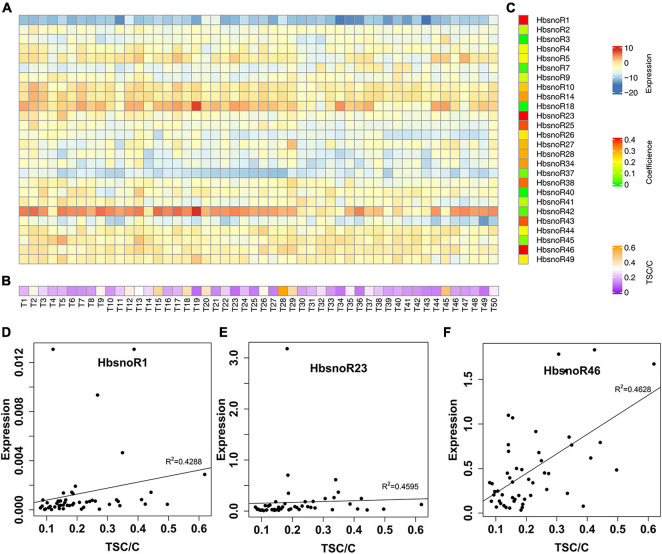
Identification of snoRNAs positively correlated with latex yield in rubber trees. **(A)** Heatmap showing the qPCR expression of 26 validated snoRNAs in a Reyan66-2 × PR107 population with 50 F1 individual trees. **(B)** Heat map of the adjusted total solid content (TSC) (TSC/C) of F1 offspring. **(C)** Heat map of the Spearman correlation coefficient for each snoRNA between expression and TSC/C in F1 offspring. A high correlation coefficient was shown in red. **(D–F)** Plots showing the correlation coefficient of *HbsnoR1*, *HbsnoR23*, and *HbsnoR46*, respectively. At least three biological replicates were used for these studies.

**FIGURE 6:**
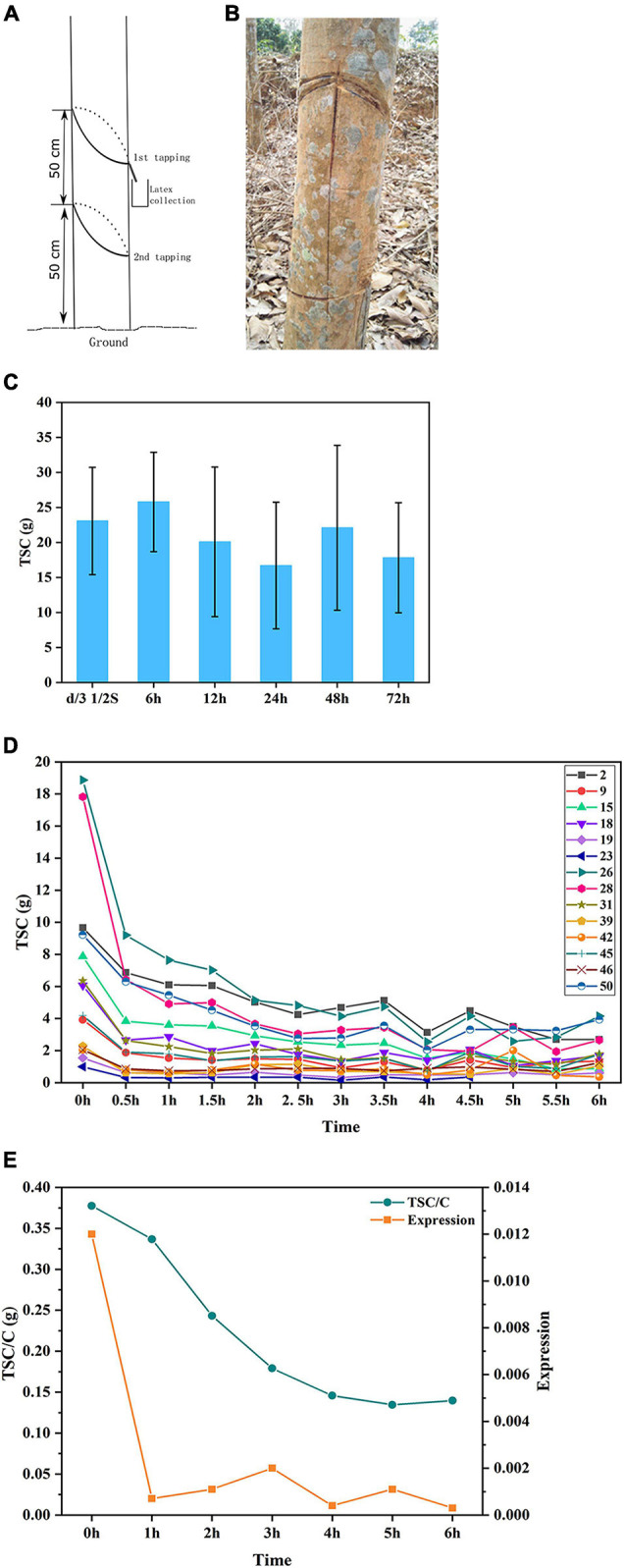
Latex regeneration study identified *HbsnoR28* as involved in latex regeneration regulation. **(A,B)** Demonstration of insulated tapping system (ITS). **(C)** Latex yield comparison between conventional S/2 d/3 tapping system and ITS with intervals of 6 to 72 h. No significant yield reduction was found for ITS 6 h. **(D)** Yield (TSC) drop trends of continuous tapping with 30-min intervals. A relatively stable TSC level was achieved after 3 h. The number denotes 14 of 50 F1 individual trees, respectively. **(E)** Profiles of *HbsnoR28* expression and TSC/C under continuous tapping with the ITS. At least three biological replicates were used for these studies.

## Results

### Nuclear RNA Sequencing and Data Analysis

To identify snoRNAs globally in the rubber tree, we constructed two cDNA libraries using the total RNAs extracted from the nuclei of the latex (Figure 1A) and leaves (Figure 1B) of the rubber tree. Then, RNA-seq was performed and 4-Gb clean paired-end reads data were obtained for each sample. A total of 27 million raw reads were obtained for the leaf samples and 21 million for the latex, with the Q30 of each sample higher than 90% (Supplementary Table 1). After filtering adapter sequences and discarding low-quality reads, the reads were mapped to the draft rubber tree genome (Liu et al., 2019). The mapping results indicated that the majority of sequences were in the exon and intergenic regions (Figure 1C), demonstrating that the RNAs extracted from the crude nuclei contained abundant amounts of mRNA and pre-mRNA. The nucleic transcriptome was then *de novo* assembled using the Trinity program (Grabherr et al., 2011). To reduce redundancy, only isoforms that had the highest expression level within each subcomponent were selected. Then, contigs that match with the rubber tree structural genes and EST sequences were removed. Finally, a total of 229,669 contigs representing unique transcripts were obtained, with an average length of 1,499 nt and N50 of 1,188 nt. These contigs were used as the ncRNA transcript repertoire from the rubber tree latex and leaf for further analysis.

### Computational Identification of Small Nucleolar RNAs

To identify snoRNA candidates, the rRNA modification sites of *2′*-*O*-methylation and pseudouridylation were first predicted according to the known sites in *A. thaliana*, *O. sativa*, and other model organisms. In total, 224 potential modification sites were identified on the rRNA sequences in the rubber tree, including 183 *2′*-*O*-methylation sites and 41 pseudouridylation sites (Table 1 and Supplementary Figure 1). These rRNA modifications were further used as the input for C/D and H/ACA subfamily snoRNA computation according to the pipeline shown in Figure 2.

**TABLE 1:** List of putative modification sites on ribosomal RNA (rRNA) sequences in *Hevea brasiliensis.*

**rRNA**	***2′*-*O*- Methylation site**	**Pseudouridylation site**
LSU	107	23
SSU	74	16
5.8S	2	2
Total	183	41

*rRNA, ribosomal RNA; LSU, large subunit; SSU, small subunit.*

For snoRNA computation, the ncRNA transcript repertoire was analyzed with the snoScan (Lowe and Eddy, 1999) and snoSeeker programs (Yang et al., 2006). A total of 22,125 snoRNA candidates were predicted, namely, 11,269 and 10,856 identified using snoScan and snoSeeker, respectively. Then, these candidate snoRNAs were subjected to a series of filtering steps (Figure 2) to remove any possible contaminations and the sequences belonging to coding genes. Subsequently, 3,626 snoRNA candidates were retained, in which 1,712 were guided C/D snoRNAs, 86 guided H/ACA snoRNAs, and 1,828 were orphan snoRNAs (Table 2).

**TABLE 2:** Statistics of identification of latex-abundant small nucleolar RNAs (snoRNAs) in *Hevea brasiliensis.*

**Analysis steps**	**snoScan (C/D guide)**	**CDSeeker**		**ACASeeker**		**Total number**
		**Guide**	**Orphan**	**Guide**	**Orphan**	
Primary prediction	11269	642	546	409	9259	22125
Removal of EST	1538	174	100	86	1728	3626
Differential expression	198	23	8	12	193	434
Removal of redundancy	172	18	7	11	147	355
High expression in latex	19	2	1	1	27	50
Semi-quantitative PCR	12	0	0	1	18	31
Related to latex regeneration	3	0	0	1	9	13

*EST, expressed sequence tag.*

### Identification of Latex-Abundant Small Nucleolar RNAs

To elucidate the potential regulatory roles of snoRNAs in latex regeneration, we analyzed the expression of snoRNAs in the latex and leaves. The expression profiles of 3,626 candidate snoRNAs were calculated and the differentially expressed (DE) snoRNAs were identified. Because of differences in the abundance of total reads between the leaves and latex samples, the pre-rRNA, which only exists in the nucleus, was used as the reference for normalization of snoRNA expression. Our results showed that 50 snoRNAs were highly expressed in latex (Figure 3A). Among the latex-abundant snoRNAs, 22 belong to the C/D subfamily and 28 belong to the H/ACA subfamily (Table 3).

**TABLE 3:** List of 50 laticifer-abundant expressed small nucleolar RNA (snoRNA) candidates in *Hevea brasiliensis.*

**Name**	**Original ID**	**Length**	**Class**	**Predicted using**	**RealFC**
*HbsnoR1*	comp134121_c0_seq4	71	C/D guide	snoScan	5273.50
*HbsnoR2*	comp135067_c1_seq25	91	C/D guide	snoScan	5142.20
*HbsnoR3*	comp133339_c1_seq1	80	C/D guide	snoScan	5110.30
*HbsnoR4*	comp127218_c0_seq2	112	C/D guide	snoScan	4702.82
*HbsnoR5*	comp134112_c0_seq38	106	C/D guide	snoScan	4036.87
*HbsnoR6*	comp138449_c0_seq9	98	C/D guide	snoScan	3803.49
*HbsnoR7*	comp136167_c0_seq2	85	C/D guide	snoScan	3440.23
*HbsnoR8*	comp127540_c0_seq7	114	C/D guide	snoScan	311.90
*HbsnoR9*	comp136053_c0_seq5	79	C/D guide	snoScan	98.57
*HbsnoR10*	comp112986_c0_seq1	105	C/D guide	snoScan	53.87
*HbsnoR11*	comp136053_c0_seq1	79	C/D guide	snoScan	44.37
*HbsnoR12*	comp55259_c0_seq1	90	C/D guide	snoScan	6.50
*HbsnoR13*	comp132131_c0_seq2	109	C/D guide	snoScan	5.73
*HbsnoR14*	comp125573_c0_seq3	129	C/D guide	snoScan	5.12
*HbsnoR15*	comp136113_c0_seq10	114	C/D guide	snoScan	4.51
*HbsnoR16*	comp132232_c0_seq2	158	C/D guide	snoScan	4.05
*HbsnoR17*	comp132541_c0_seq6	76	C/D guide	snoScan	3.57
*HbsnoR18*	comp131062_c0_seq3	103	C/D guide	snoScan	3.56
*HbsnoR19*	comp135337_c0_seq2	85	C/D guide	snoScan	3.04
*HbsnoR20*	comp130910_c0_seq1	70	C/D guide	CDSeeker	2.85
*HbsnoR21*	comp138599_c0_seq5	143	C/D guide	CDSeeker	2.84
*HbsnoR22*	comp123171_c0_seq1	130	orphan	CDSeeker	4.93
*HbsnoR23*	comp127086_c0_seq5	129	H/ACA guide	ACASeeker	12485.31
*HbsnoR24*	comp138286_c0_seq14	157	orphan	ACASeeker	14668.81
*HbsnoR25*	comp83639R_c0_seq3	145	orphan	ACASeeker	8562.99
*HbsnoR26*	comp125664_c0_seq1	161	orphan	ACASeeker	5303.07
*HbsnoR27*	comp86093_c0_seq1	122	orphan	ACASeeker	4788.97
*HbsnoR28*	comp117226_c0_seq1	137	orphan	ACASeeker	4759.08
*HbsnoR29*	comp133438_c0_seq17	145	orphan	ACASeeker	4527.53
*HbsnoR30*	comp135975_c0_seq12	131	orphan	ACASeeker	3567.82
*HbsnoR31*	comp89929_c0_seq2	142	orphan	ACASeeker	2788.92
*HbsnoR32*	comp136348_c1_seq1	124	orphan	ACASeeker	353.33
*HbsnoR33*	comp132848_c0_seq1	138	orphan	ACASeeker	226.49
*HbsnoR34*	comp113290_c0_seq3	121	orphan	ACASeeker	77.61
*HbsnoR35*	comp129581_c0_seq2	147	orphan	ACASeeker	60.00
*HbsnoR36*	comp132931_c0_seq2	122	orphan	ACASeeker	22.58
*HbsnoR37*	comp129581_c0_seq3	147	orphan	ACASeeker	17.36
*HbsnoR38*	comp115823_c0_seq1	125	orphan	ACASeeker	12.87
*HbsnoR39*	comp141150_c0_seq1	142	orphan	ACASeeker	10.58
*HbsnoR40*	comp137921_c0_seq3	143	orphan	ACASeeker	9.69
*HbsnoR41*	comp136512_c0_seq1	157	orphan	ACASeeker	8.06
*HbsnoR42*	comp136486_c0_seq1	141	orphan	ACASeeker	6.90
*HbsnoR43*	comp134776_c0_seq2	140	orphan	ACASeeker	5.84
*HbsnoR44*	comp137994_c0_seq3	135	orphan	ACASeeker	5.14
*HbsnoR45*	comp135572_c0_seq1	143	orphan	ACASeeker	4.97
*HbsnoR46*	comp137742_c0_seq1	149	guide	ACASeeker	4.44
*HbsnoR47*	comp131146_c0_seq2	152	orphan	ACASeeker	3.45
*HbsnoR48*	comp134726_c0_seq6	139	orphan	ACASeeker	3.44
*HbsnoR49*	comp135184_c1_seq2	156	orphan	ACASeeker	3.26
*HbsnoR50*	comp136849_c0_seq1	144	orphan	ACASeeker	2.41

Semi-quantitative RT-PCR was performed to verify the expression levels of laticifer-abundant expressed snoRNAs identified from RNA-seq data analysis. The pre-rRNA transcript was used as the reference gene because mature rRNAs are not present in the nucleus. The amplification fragment of pre-rRNAs was designed to span the internal transcribed spacer 1 (ITS1). Among the 50 latex-abundant snoRNAs, 31, including 12 C/D snoRNAs (12 guides) (Figure 3B) and 19 H/ACA snoRNAs (1 guide, 18 orphans) (Figure 3C) were highly expressed in laticifer compared with leaves (Table 2). The RT-PCR results further confirmed that these snoRNA were present abundantly in the latex (Figure 3).

### Prediction of the Target Sites of Latex-Abundant Small Nucleolar RNAs

To elucidate the functions of snoRNAs in the rubber tree, we predicted their potential target sites according to sequence recognition rules. Previous studies have indicated that snoRNAs have a stable secondary structure and that their modification sites (*2′*-*O*-methylation sites and pseudouridylation sites) are usually conserved in eukaryotes, such as *A. thaliana*, *O. sativa*, and other model organisms (Watkins and Bohnsack, 2012; Dupuis-Sandoval et al., 2015). The 13 latex-abundant guide snoRNAs (12, C/D subfamily member and 1, H/ACA subfamily member) were found to target 31 specific modification sites (Figure 4). For 18S rRNAs, there are 12 modification sites that are targeted by seven-latex abundant snoRNAs, whereas for 28S rRNAs, the numbers are 19 and 10. Except *HbsnoR23*, which was predicted to target the pseudouridylation of U2790 of 28S rRNA, all the other 12 snoRNAs belong to the C/D subfamily and direct the *2′*-*O*-methylation modification of 30 sites on 18S and 28S rRNAs (Table 4).

**TABLE 4:** Targeted modification sites of 13 latex-abundant guide snoRNA on 18S and 28S rRNAs.

**snoRNAs**	**18S rRNA**	**28S rRNA**
	***2′*-*O*-methylation site**	
*HbsnoR1*	U791	–
*HbsnoR2*	A265, G1074, G1374, and A1749	G2041, G2079, U2090, and G3252
*HbsnoR3*	–	C1060
*HbsnoR5*	–	A2808
*HbsnoR6*	–	C111
*HbsnoR7*	A1193 and A1423	–
*HbsnoR8*	A203	–
*HbsnoR11*	C242	C865, A1664, and U2041
*HbsnoR12*	A1417	A1482
*HbsnoR13*	–	U2798, A2801, and A3149
*HbsnoR15*	U1539, U1545	A1512, U2868, and U2900
*HbsnoR19*	–	G301
	**Pseudouridylation**	
*HbsnoR23*	–	U2790

### Identification of Small Nucleolar RNAs Involved in Regulating Latex Regeneration

To identify snoRNAs that are related to latex yield potential, a correlation analysis was conducted between the latex yield and the expression of snoRNAs in an F1 population of Reyan66-2 × PR107. We randomly selected a total of 50 F1 individual trees, and the adjusted yields were ranked. In the rubber tree, a larger girth means a longer tapping cut and usually produces more latex, because more laticifers are present in the bark with a larger circumference. Therefore, we estimated the potential of latex production by normalizing the TSC by dividing with the trunk circumference (TSC/C), which will avoid the estimation bias caused by differences in trunk size. A Spearman correlation was performed to assess the relationship between the expression of snoRNAs (Figure 5A) and the ranked TSC/C (Figure 5B). The results showed that 13 of the 26 laticifer-abundant snoRNAs were positively correlated with latex yield potential (TSC/C) (*R*^2^ > 0.2, Figure 5C and Supplementary Table 3). Among the snoRNAs that were correlated with latex yield, *HbsnoR1*, *HbsnoR23*, and *HbsnoR46* showed high correlation coefficients (*R*^2^ > 0.4, Figures 5D–F). *HbsnoR23* and *HbsnoR1* belong to the guide H/ACA subfamily, while *HbsnoR46* is an orphan H/ACA snoRNA. This result suggested that orphan H/ACA snoRNAs might participate in the regulation of latex production through pathways other than guiding rRNA modifications.

The rubber tree laticifers are connected as a network structure that enables the latex to flow for a long distance when the bark is tapped. Many factors influence the movement of the latex in the laticifer network, such as laticifer turgor and plugging index. To minimize the replenish effect of long-distance movement of the latex, a 50-cm-tall bark was insulated by girding the trunk at a height of 50 and 100 cm. The girding was carefully conducted to cut the yellow layer, where the laticifers are abundant while causing no damage to the inner watery layer, in which the nutrients are transported (Figures 6A,B). Thus, this ITS will exude the latex only from the dedicated 50-cm bark, and the transportation of the nutrients will not be affected.

First, we evaluated the minimum time required for latex regeneration in the rubber tree. We selected five individual trees for tapping using the ITS. The trees were continuously tapped at intervals of 6, 12, 24, 48, and 72 h, and the TSC was calculated. The results showed that latex can be completely regenerated as quickly as 6 h (Figure 6C). To further investigate the dynamics of latex regeneration, the laticifers in the ITS were isolated by continuous tapping until no latex exuded. After 12 h of regeneration, the trees were tapped at 30-min intervals, and the TSC was calculated. The results indicated that the TSC at each tapping dropped sharply in the first six tappings and then reached a stable state (Figure 6D). After 3 h, the TSC of each tapping was maintained at a relatively stable level, suggesting that it was regenerated in the previous 30 min. We, therefore, considered the TSC at 3 h as the latex regeneration ability and investigated the correlation with snoRNA expression at this time point. We investigated the expression of 13 snoRNAs positively correlated with latex yield by Q-PCR and calculated their correlation with adjusted TSC (TSC/C). The results showed that one snoRNA (*HbsnoR28*) displayed a high correlation coefficient (*R*^2^ = 0.6039) with TSC/C (Table 5). The examination of *HbsnoR28* expression showed that its expression was downregulated and highly consistent with TSC/C during continuous tapping, suggesting the regulatory roles of this snoRNA in latex regeneration (Figure 6E).

**TABLE 5:** The Spearman correlation coefficient between the expression of 13 latex-yield-related small nucleolar RNAs (snoRNAs) and latex regeneration ability.

**snoRNA**	**Correlation coefficient**
*HbsnoR28*	0.6039
*HbsnoR43*	0.0905
*HbsnoR14*	0.0707
*HbsnoR49*	0.0418
*HbsnoR26*	−0.0117
*HbsnoR46*	−0.0861
*HbsnoR1*	−0.1703
*HbsnoR38*	−0.2110
*HbsnoR27*	−0.2165
*HbsnoR23*	−0.2616
*HbsnoR10*	−0.3152
*HbsnoR25*	−0.3386
*HbsnoR34*	−0.3586

## Discussion

Compared with studies on snoRNA in animals, those on snoRNAs in plants have not been extensively performed. Previously, 295 and 544 snoRNAs have been identified in plants such as *A. thaliana* (Kim et al., 2010) and *O. sativa* (Liu et al., 2012), respectively. *H. brasiliensis* is one of the most important industrial crops; however, a snoRNA has not been extensively studied in *H. brasiliensis* thus far. To date, only five snoRNAs have been annotated in a microRNA transcriptome from the leaf, bark, and root tissues (Gébelin et al., 2012). NcRNA sequencing techniques are powerful tools for the identification of snoRNAs. The RNA sequencing of the *H. brasiliensis* ncRNA library produced 229,569 sequences, and 3,626 of them were putative snoRNA candidates. After a further DE expression analysis and RT-PCR validation, 31 of them were identified to be latex-abundant snoRNAs. To the best of our knowledge, the global investigation of laticifer snoRNAs in the rubber tree have been reported for the first time in this study, and our results may be very important in understanding the mechanism underlying latex metabolism and regeneration.

The functions of canonical snoRNAs are guiding the modification of rRNAs, snRNAs, tRNAs, or known stable RNA molecules. The most prevalent modifications found in rRNA molecules are the methylation of the ribose moiety at the *2′*-hydroxyl group (*2′*-*O*-methylation) and the conversion of uridine to pseudouridine (Ψ). In this study, 31 putative modification sites were targeted by 13 laticifer-abundant guide snoRNAs in *H. brasiliensis*. Modification sites on the rRNA are mostly concentrated on the functionally important regions of the ribosome according to the 3D structural map, which would influence the activities of the ribosome. The putative modification sites on rRNA in *H. brasiliensis* may provide a clue for understanding the mechanism of how snoRNAs direct the modification of rRNA and regulate latex regeneration. In addition, 18 laticifer-abundant orphan snoRNAs were identified. Currently, no target RNA molecules are reported; however, it could be suggested that they might be involved in the regulation of physical processes, such as RNA silencing, rRNA chaperones, alternative splicing, and chromatin packaging.

To date, a limited number of studies have investigated snoRNA functions associated with physiological processes in plants. Loss of three snoRNAs (U33, U34, and U35a) in the *rpL13a* locus was sufficient to induce resistance to oxidative and lipotoxic stresses *in vitro* and prevented the spread of oxidative stress *in vivo* (Michel et al., 2011). In the conventional tapping system, large amounts of proteins and rRNAs are lost with the latex exudate and need to be replenished before the next tapping. rRNA was the first to be synthesized and is required for peptide translation. The regeneration of RNA will directly affect latex regeneration. Our results showed that 13 snoRNAs were positively correlated with latex production. Among these positively correlated snoRNAs, *HbsnoR28* (H/ACA subfamily) showed a high correlation (*R*^2^ = 0.6039) with latex regeneration potential in our ITS. This 138-nt-long orphan H/ACA snoRNA is novel and displays no homology with known snoRNAs in other species. Characterization of the functions of snoRNAs will be of great significance to examine latex metabolism and regeneration.

In this study, we developed a novel ITS, which separates the laticifers in dedicated regions from the laticifer network of the whole trees. The purpose of the ITS is to eliminate the long distance transportation of the latex in the laticifer network, which greatly influences the estimation of latex regeneration potential because many factors influence latex flow during the tapping process. This newly developed tapping system may cause some damage to the tree, and cannot be implemented in the latex harvest process for farmers. However, it has great advantages for studying the functions of laticifers and latex metabolism and regeneration, as the ITS simplified factors that influence latex biosynthesis and regeneration. We suggest that this system may have a great application significance for fundamental studies on laticifer biology and physiology.

## Conclusion

In summary, non-coding RNA and transcriptome libraries were constructed in the rubber tree leaf and latex. We performed high-throughput sequencing to identify a large number of snoRNAs in the rubber tree, and 31 snoRNAs were confirmed to be highly expressed in latex. Further functional analysis of these snoRNAs will be useful for a better understanding of the complexity and dynamics of latex regeneration in the rubber tree. Thus, our findings might provide a valuable foundation for exploring the snoRNA-mediated regulatory mechanism involved in latex metabolism and regeneration.

## Data Availability Statement

The original contributions presented in the study are publicly available. This data can be found here: National Center for Biotechnology Information (NCBI) BioProject database under accession number PRJNA742874.

## Author Contributions

HC coordinated the project and supervised the data generation and analysis. XC and ZD conducted most of the experiments and wrote the draft of the manuscript. XZ and XH maintained the rubber tree population and collected the tapping data. DY, WW, ZA, and QL partially performed the field experiments and discussed the manuscript. HH helped to design the whole project. All authors reviewed and approved the final manuscript.

## Conflict of Interest

The authors declare that the research was conducted in the absence of any commercial or financial relationships that could be construed as a potential conflict of interest.

## Publisher’s Note

All claims expressed in this article are solely those of the authors and do not necessarily represent those of their affiliated organizations, or those of the publisher, the editors and the reviewers. Any product that may be evaluated in this article, or claim that may be made by its manufacturer, is not guaranteed or endorsed by the publisher.

## Footnotes

1
https://gist.github.com/zachcp/c45433307a292513af41

## Supplementary Material

Supplementary Figure 1 Predicted modification sites in 5.8S, 18S, and 28S rRNA sequences in *Hevea brasiliensis*.

Supplementary Figure 2 Spearman correlation coefficient between the expression of 10 laticifer-abundant small nucleolar ribonucleic acids (snoRNAs) and the total latex production and total solid content (TSC)/C (*R*^2^ = 0.2–0.4).

Supplementary Table 1 Sequences analysis of snoRNA libraries by high-throughput sequencing.

Supplementary Table 2 Putative snoRNA candidates in rubber tree.

Supplementary Table 3 Spearman correlation coefficient between the expression of 31 laticifer-abundant snoRNAs and the total latex production and TSC/C.

Supplementary Table 4 Primers were used for quantitative reverse transcription polymerase chain reaction (qRT-PCR) in this study.
