# Supplementary figures and images for: Genome-Wide Identification and Analysis of Small Nucleolar RNAs and Their Roles in Regulating Latex Regeneration in the Rubber Tree (Hevea brasiliensis)
Source: Front Plant Sci. 2021 Oct 26;12:731484. doi: 10.3389/fpls.2021.731484 (PMC8575768; doi:10.3389/fpls.2021.731484)

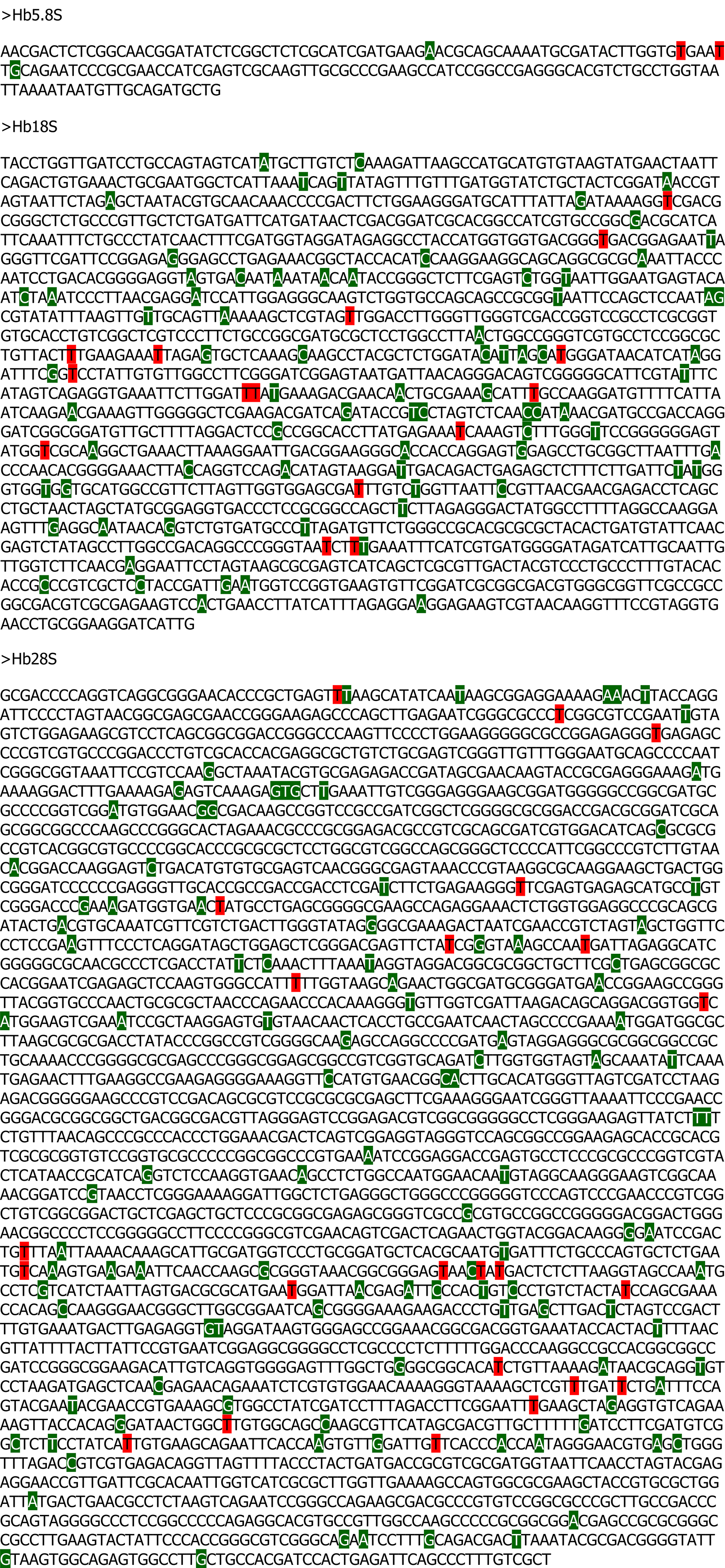

Supplement: Supplementary Figure 1 — Predicted modification sites in 5.8S, 18S, and 28S rRNA sequences in Hevea brasiliensis. [file Image_1.TIF]

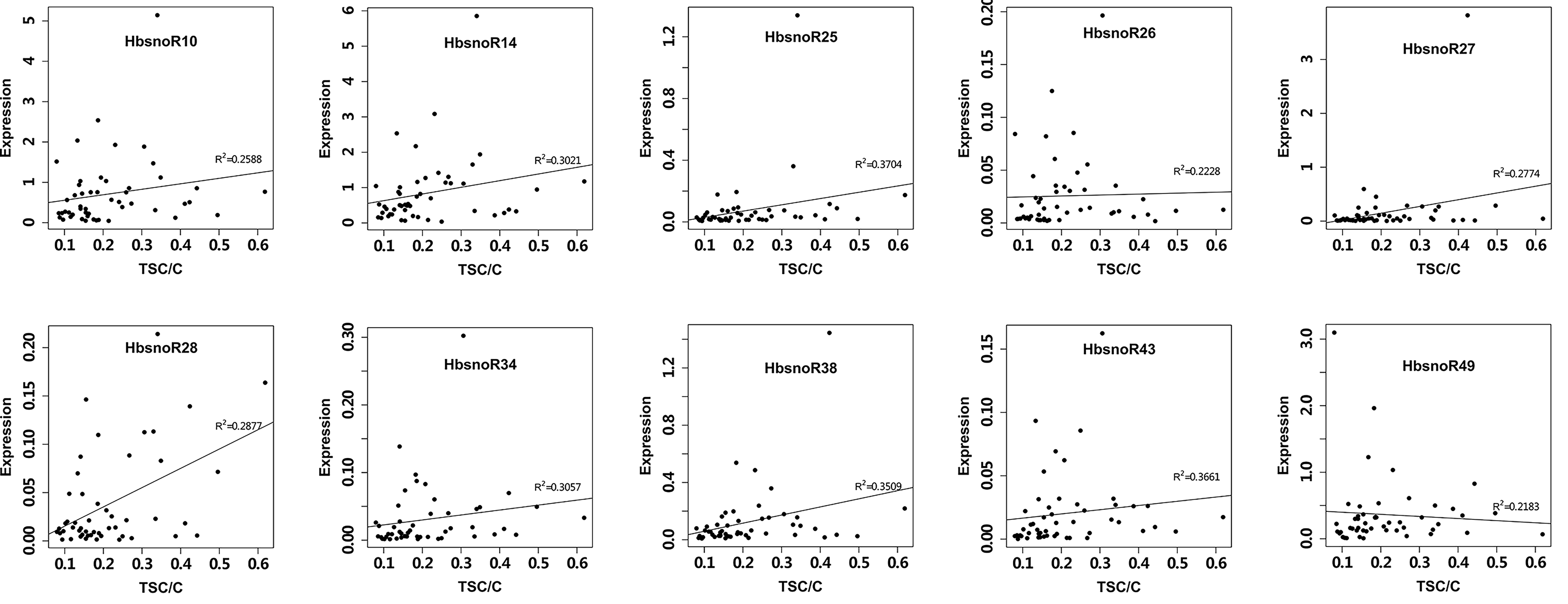

Supplement: Supplementary Figure 2 — Spearman correlation coefficient between the expression of 10 laticifer-abundant small nucleolar ribonucleic acids (snoRNAs) and the total latex production and total solid content (TSC)/C (R2 = 0.2–0.4). [file Image_2.TIF]
